# Myocardial production and release of MCP-1 and SDF-1 following myocardial infarction: differences between mice and man

**DOI:** 10.1186/1479-5876-9-150

**Published:** 2011-09-12

**Authors:** Andrew J Boyle, Yerem Yeghiazarians, Henry Shih, Joy Hwang, Jianqin Ye, Rich Sievers, Daiwei Zheng, Jath Palasubramaniam, Dharshan Palasubramaniam, Connie Karschimkus, Robert Whitbourn, Alicia Jenkins, Andrew M Wilson

**Affiliations:** 1 Department of Medicine, Division of Cardiology, University of California San Francisco, San Francisco USA; 2 St Vincent's Hospital Department of Medicine, University of Melbourne, Melbourne Australia

## Abstract

**Background:**

Stem cell homing to the heart is mediated by the release of chemo-attractant cytokines. Stromal derived factor -1 alpha (SDF-1a) and monocyte chemotactic factor 1(MCP-1) are detectable in peripheral blood after myocardial infarction (MI). It remains unknown if they are produced by, and released from, the heart in order to attract stem cells to repair the damaged myocardium.

**Methods:**

Murine hearts were studied for expression of MCP-1 and SDF-1a at day 3 and day 28 following myocardial infarction to determine whether production is increased following MI. In addition, we studied the coronary artery and coronary sinus (venous) blood from patients with normal coronary arteries, stable coronary artery disease (CAD), unstable angina and MI to determine whether these cytokines are released from the heart into the systemic circulation following MI.

**Results:**

Both MCP-1 and SDF-1a are constitutively produced and released by the heart. MCP-1 mRNA is upregulated following murine experimental MI, but SDF-1a is suppressed. There is less release of SDF-1a into the systemic circulation in patients with all stages of CAD including MI, mimicking the animal model. However MCP-1 release from the human heart following MI is also suppressed, which is the exact opposite of the animal model.

**Conclusions:**

SDF-1a and MCP-1 release from the human heart are suppressed following MI. In the case of SDF-1a, the animal model appropriately reflects the human situation. However, for MCP-1 the animal model is the exact opposite of the human condition. Human observational studies like this one are paramount in guiding translation from experimental studies to clinical trials.

## Background

The adult mammalian heart has limited capacity for self-repair. Enhancing the heart's own reparative mechanisms represents an attractive therapeutic option for patients who have suffered myocardial infarction (MI). Stem cell chemo-attractant cytokines are thought to be released from the heart in response to injury, in an attempt to stimulate endogenous tissue repair pathways. Monocyte chemotactic protein-1 (MCP-1, CCL2) and stromal-derived factor 1 alpha (SDF-1a, CXCL12) are two stem cell chemo-attractant cytokines that are thought to play a role in post-infarct cardiac repair.

SDF-1a is constitutively expressed by many tissues, including the heart [1]. Following MI, cardiac SDF-1a is thought to contribute to a stem cell cytokine gradient that causes homing of stem cells to the heart [2,3]. For this to occur, SDF-1a must be produced in the heart and released into the circulation. Experimental models have yielded conflicting data about cardiac expression of SDF-1a, with some finding increased expression after MI [2,4-6] and others finding decreased expression [1,7]. Furthermore, the data concerning serum levels of circulating SDF-1a are also conflicting. In patients, some studies find increased levels after MI [8,9], while others find no change [10]. In rodent experimental MI, the serum level of SDF-1a is actually reduced [2], whereas in the canine model it is increased [1]. Thus, whether SDF-1a is released from the heart to create a cytokine gradient following MI remains unknown.

The role of MCP-1 in post-infarction left ventricular (LV) remodeling is complex. MCP-1 has been implicated in numerous steps along the way to post-infarction heart failure: in the development of atherosclerosis [11], in atherosclerotic plaque instability [12], in recruitment of monocytes to the heart following MI [13] and in post-infarction LV remodeling [14]. In experimental animal models, MCP-1 is also chemoattractant for stem cells [15,16]. MCP-1 levels are increased in peripheral blood following MI, both in rats [17] and in patients [18-20] and these levels correlate with prognosis [21]. It has been assumed that these peripheral blood levels are reflective of cardiac release causing a chemo-attractant cytokine gradient towards the heart. Thus modulation of the MCP-1/CCR2 axis has been proposed as a novel therapeutic target to treat post-infarction LV remodeling [14,21].

It is not clear whether the serum levels of these cytokines after MI is reflective of cardiac production and release, or whether they are coming from other tissues. Before clinical trials utilizing these cytokines as potential therapeutic agents are undertaken, these questions must be addressed. We therefore have studied the murine and human patterns of myocardial production and release of these cytokines in response to MI.

## Methods

### Animal study

Male C57BL/6J were used for all murine experiments. Animals were handled according to the guidelines of the Institutional Animal Care and Use Committee at the University of California San Francisco.

#### Myocardial infarction

MIs were induced surgically by a permanent ligation of the left anterior descending (LAD) coronary artery as previously described [22]. Briefly, after intubation and ventilation, thoracotomy is performed and permanent ligation of the LAD is made by a 7-0 suture in the anterior myocardium at 50% of the length of the heart from the anterior-inferior edge of the left atrium to the apex.

#### Tissue preparation and analysis

Animals were humanely sacrificed under general anesthesia. Baseline non-infarcted animals (n = 3) were compared with post-infarct animals at 2 time-points following MI: early (day 3; n = 3) and late (day 28; n = 3). Hearts were arrested in diastole with injection of concentrated KCl and the hearts immediately removed. For RNA analysis, the left ventricle was dissected away from the rest of the heart, divided into infarct, border and remote zones, and rapidly frozen by immersion in liquid nitrogen. Total RNA was isolated from left ventricles by TRIzol reagent (Invitrogen). Trace genomic DNA in total RNA was removed by Dnase I and Rneasy Mini Kit (Qiagen). cDNA was generated from 0.1 mg of total RNA by using SuperScript III First-Strand Synthesis kit (Invitrogen). Microarrays were performed using Affymetrix 1.0 Mouse gene chip. Data were normalized using robust multi-array average (RMA) method. Control and low performing probesets (those with intensity values below a threshold across all samples, the threshold was taken to be the global lowest 25^th ^percentile of intensity values) were excluded from analysis. Also, based on Affymetrix's annotation information, only those probesets which were part of the main design of the array and perfectly matching only one sequence were considered for analysis of differential expression. 20648 out of 35557 probesets remained after filtering

### Human Study

The human clinical study was approved by the Human Research Ethics Committee at St Vincent's Hospital Melbourne Australia. Patients undergoing cardiac catheterization or cardiac surgery gave written informed consent for blood samples to be taken from the coronary artery/aortic root and coronary sinus. For the cardiac catheterization patients, blood was taken from the coronary artery ostium via the cardiac catheter used for the case. A catheter was placed into the coronary sinus under fluoroscopic guidance and a sample was taken from the sinus at the same time. For the cardiac surgery patients, during cannulation of the heart in preparation for bypass, blood samples were taken from the coronary sinus (retrograde cardioplegia catheter) and from the aortic root (aortic bypass cannula). Importantly, in order to eliminate white blood cell activation from the bypass pump confounding our results, samples were taken before initiation of cardiopulmonary bypass and the blood had not entered the pump circuit. The blood was separated into serum and plasma and stored at -80 degrees C. For cytokine levels, ELISA was performed using antibodies to MCP-1 and SDF-1a R and D systems, Minneapolis MN) according to the manufacturer's protocol. The degree of coronary artery disease was assessed by an experienced cardiologist who was blinded to the results of the serum analysis. Patients were placed into categories depending on the degree of coronary artery disease, the clinical presentation and the results of serum cardiac enzymes and electrocardiogram (ECG). Patients were then described as angiographically normal coronary arteries, stable coronary artery disease, unstable angina and acute MI.

### Statistical analysis

For murine mRNA studies, moderated t-statistics (as implemented in the limma package in R/Bioconductor) were used to test for differentially expressed probesets. The theoretical (raw) p-values were then adjusted for multiple testing by controlling the false discovery rate (FDR). A cut-off of 0.05 was used on the FDR adjusted p-values to declare a probeset to be significant. For human studies, data are presented as mean ± SD unless otherwise stated. Cytokine levels are compared between groups using ANOVA with post hoc Fishers test. Patient demographics were compared using one-way ANOVA with Bonferroni post-hoc correction for continuous variables (age), and Pearson's Chi-square for comparison of categorical variables for other demographic data. A p-value of 0.05 or less is considered significant.

## Results

### Myocardial Production and Release of SDF-1a are Similar in Murine and Human Myocardial Infarction Respectively

**Figure 1:**
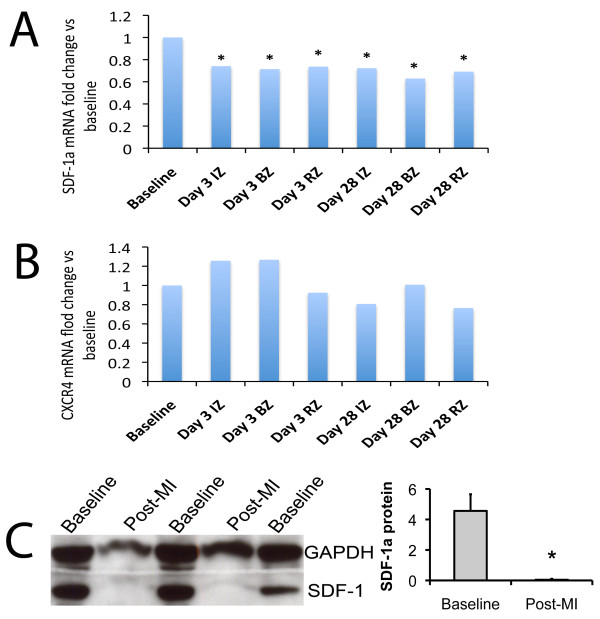
**SDF-1a expression in the murine heart**. There is reduced expression of mRNA for SDF-1a (A) in all regions of the murine heart following myocardial infarction. This occurs early, and remains suppressed for 28 days. There is no change in mRNA for its receptor CXCR4 (B). (C) Myocardial protein levels of SDF-1a are also reduced following MI. IZ = infarct zone; BZ = border zone of the infarct; RZ = remote zone. n = 3 for each region at each time-point.

In the adult mammalian heart, mRNA encoding for SDF-1a and its cognate receptor CXCR4 are detectable at baseline. Following myocardial infarction, the constitutive expression of SDF-1a mRNA is reduced by approximately 25% at day 3, and this persists out to day 28 in all regions of the heart (Figure 1A). The expression of CXCR4, the receptor for SDF-1a, is unchanged in any region of the heart at either time-point following MI (Figure 1B). Similarly, the protein level of SDF-1a is reduced following MI (Figure 1C).

**Figure 2:**
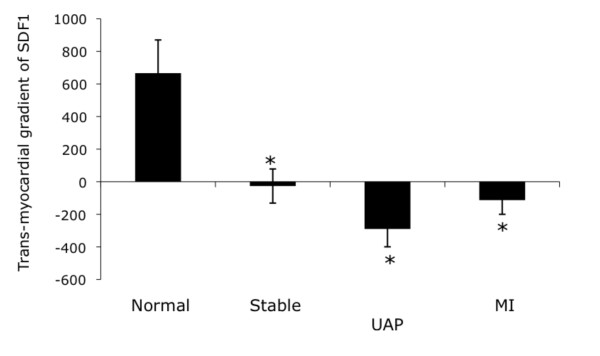
**SDF-1a release from the human heart**. In humans, SDF-1a is constitutively released from hearts with normal coronary arteries. However, following MI, myocardial release of SDF-1a is reduced. This is consistent with the murine mRNA expression pattern. SDF-1a release is suppressed in all stages of coronary artery disease, suggesting that coronary artery disease, rather than the tissue damage from MI, is responsible for the suppression of SDF-1a release. Transmyocardial gradient = coronary sinus level - coronary artery level; stable = stable coronary artery disease; UAP = unstable angina pectoris; MI = myocardial infarction.

In humans, SDF-1a is constitutively released from the heart into the circulation at baseline, resulting in a positive gradient from the coronary artery to the coronary sinus (Table 1, Figure 2) and this release is suppressed after MI. Furthermore, SDF-1a release is also abolished in stable coronary artery disease and in unstable angina, suggesting that the presence of coronary artery disease, rather than the myocardial damage during MI, is responsible for the reduction in cardiac release of SDF-1a. The changes in cardiac production and release of SDF-1a in response to MI appear to be similar in the mouse and human.

**Table 1:** Cytokine Levels in Patients With Coronary Artery Disease

	Coronary Artery	Coronary Sinus	Gradient
**MCP1**			
**Normal**	94.6 ± 54.1	146.7 ± 56.3	52.1 ± 24.3
**Stable CAD**	104.9 ± 46.7	116.7 ± 52.2	11.7 ± 21.9 *
**UAP**	65.3 ± 27.5	79.4 ± 18.4 *	14.1 ± 12.2 *
**MI**	87.8 ± 41.4	101.9 ± 38.2 *	12.5 ± 28.4 *
**SDF-1a**			
**Normal**	1982.0 ± 608.1	2648.3 ± 896.3	666.3 ± 288.1
**Stable CAD**	2570.8 ± 694.4	2544.3 ± 710.8	-26.4 ± 377.7 *
**UAP**	1694.0 ± 321.6	1404.0 ± 444.2 *	-290.0 ± 244.8 *
**MI**	1705.4 ± 298.3	1592.4 ± 350.4 *	-113.0 ± 325.6 *

* p < 0.05 vs Normal. CAD = coronary artery disease; UAP = unstable angina pectoris; MI = myocardial infarction.

### Myocardial Production and Release of MCP-1 Differ Between Murine and Human Myocardial Infarction

**Figure 3:**
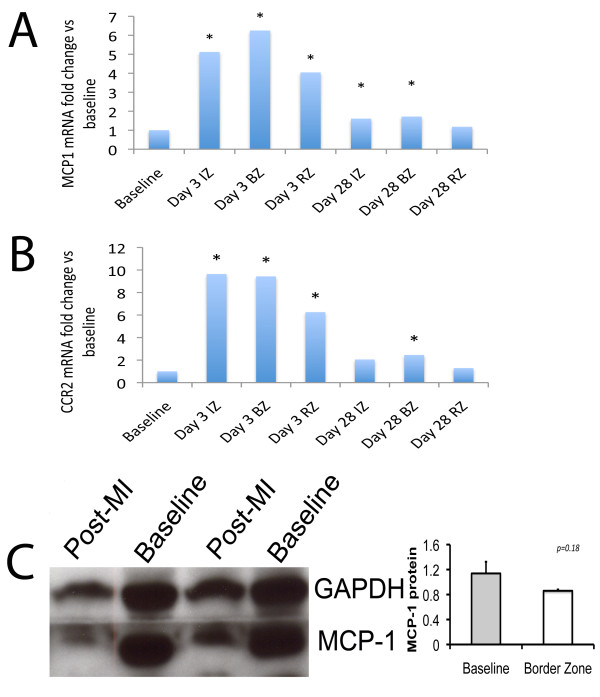
**MCP-1 expression in the murine heart**. Increased expression of mRNA for MCP1 (A) and its cognate receptor CCL2 (B) in the murine heart following myocardial infarction occurs early, then returns to near baseline levels after 28 days. (C) MCP-1 protein levels are not statistically significantly different following MI. IZ = infarct zone; BZ = border zone of the infarct; RZ = remote zone.

At 3 days after MI, MCP1 mRNA increases 4-6 fold in all regions of the heart, and returns toward baseline by day 28 (Figure 3A). This time-dependent rise in the expression of the ligand is mirrored by its receptor CCR2 (Figure 3B). The protein level of MCP-1 is not increased in mice following MI (Figure 3C).

**Figure 4:**
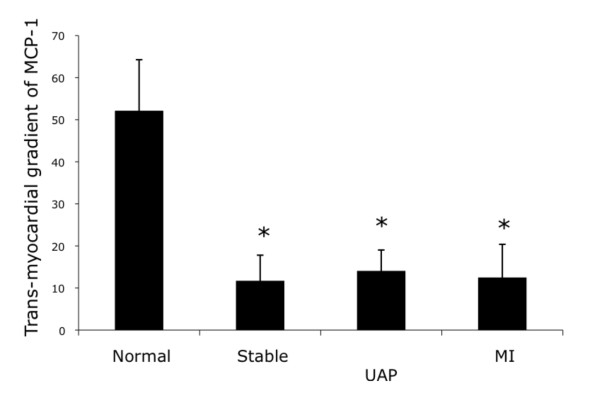
**MCP-1 release from the human heart**. In humans, MCP-1 is constitutively released from hearts with normal coronary arteries. However, following myocardial infarction, myocardial release of MCP-1 is reduced. This is the opposite pattern suggested from the murine mRNA studies. Furthermore, MCP-1 release is suppressed in all stages of coronary artery disease, suggesting that coronary artery disease, rather than the tissue damage from MI, is responsible for the suppression of MCP-1 release. Transmyocardial gradient = coronary sinus level - coronary artery level; stable = stable coronary artery disease; UAP = unstable angina pectoris; MI = myocardial infarction.

In humans, MCP-1 is constitutively released from the heart at baseline (Table 1). However, in direct contrast to the murine model, MCP-1 release from the heart is suppressed following myocardial infarction (Figure 4, Table 1). Furthermore, MCP-1 release is suppressed in stable CAD and unstable angina to similar degrees as following MI, suggesting that the presence of coronary artery disease, rather than the myocardial damage during MI, is responsible for the reduction in cardiac release of MCP-1. The changes in cardiac production and release of MCP-1 in response to MI appear to be the opposite in the mouse and human.

### Infarct Size Does Not Affect Human Myocardial Cytokine Release

Transmyocardial gradient of MCP-1 and SDF-1a had no correlation with infarct size, as determined by peak troponin-I (TnI) level or peak creatinine kinase (CK) level. Pearson's correlation *r*-value between TnI and MCP-1 was 0.02 (p = 0.95) and between TnI and SDF-1a was 0.31 (p = 0.30); between peak CK and MCP-1 was -0.06 (p = 0.88) and between CK and SDF-1a was 0.31 (p = 0.38).

### Influence of Timing and Patient Demographics After MI

**Figure 5:**
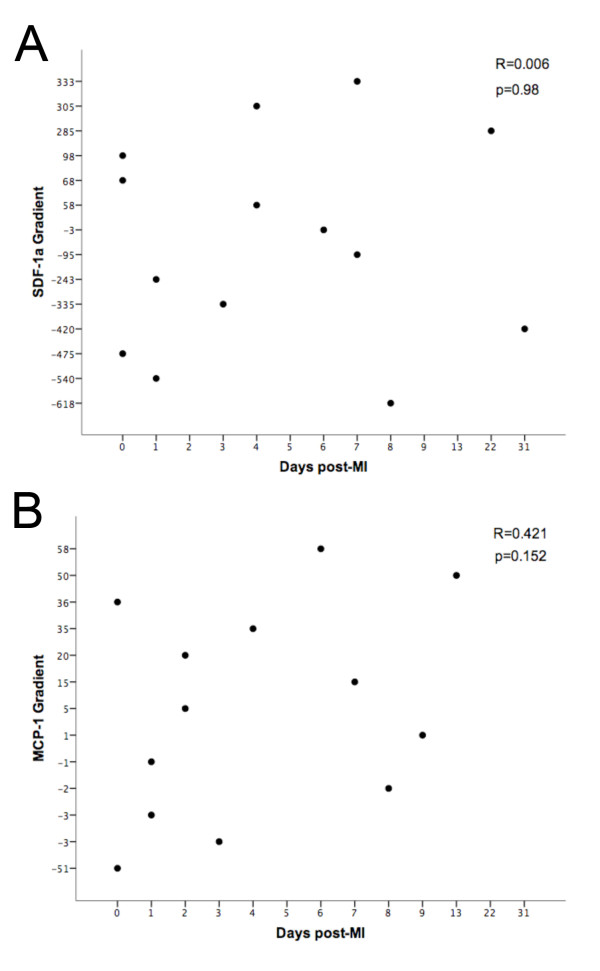
**Timescale of cytokine release following MI**. There is no correlation between the trans-myocardial gradient of SDF-1a (A) or MCP-1 (B) with the time from infarction. R = Pearson's correlation co-efficient.

To ensure that the time post-MI that the samples were taken did not influence our results, we plotted the trans-myocardial gradients of SDF-1a and MCP-1 versus time from the MI. The trans-myocardial gradients of SDF-1a and MCP-1 were not related to the time measured after MI (Figure 5). Furthermore, the patients' age, risk factors and medications were no different between groups. There was, however, a significantly higher percentage of males in the coronary artery disease groups compared to the normal controls (Table 2).

**Table 2:** Patient demographics

		Normal	Stable CAD	Unstable Angina	MI
		n = 4	n = 15	n = 6	n = 21
Male*	*n (%)*	1 (25%)	10 (67%)	5 (83%)	19 (90%)
Age (years)	*Mean +/- s.d*	65 +/- 0.5	70 +/- 8.1	58 +/- 11	61 +/- 12

**Cardiovascular Risk Factors**					
Diabetes Mellitus	*n (%)*	2 (50%)	6 (40%)	1 (17%)	11 (52%)
Hypertension	*n (%)*	4 (100%)	12 (80%)	3 (50%)	13 (62%)
Dyslipidemia	*n (%)*	2 (50%)	12 (80%)	4 (67%)	16 (76%)
Family Hx	*n (%)*	0 (0%)	11 (73%)	4 (67%)	12 (57%)
Smoking Hx	*n (%)*	1 (25%)	7 (47%)	4 (67%)	15 (71%)

**Medications**					
Nitroglycerin	*n (%)*	1 (25%)	5 (33%)	4 (67%)	7 (33%)
Statin	*n (%)*	1 (25%)	7 (47%)	3 (50%)	14 (67%)
ACEI/ARB	*n (%)*	2 (50%)	8 (53%)	2 (33%)	12 (57%)
Beta Blocker	*n (%)*	1 (25%)	6 (40%)	5 (83%)	13 (62%)
Aspirin	*n (%)*	2 (50%)	9 (60%)	3 (50%)	16 (76%)

*p < 0.05. CAD = coronary artery disease; MI = myocardial infarction; Hx = history; ACEI = angiotensin converting enzyme inhibitor; ARB = angiotensin receptor blocker.

## Discussion

This study has several important novel findings. Firstly, myocardial release of SDF-1a into the circulation is decreased in humans following MI and in all stages of coronary artery disease. Secondly, myocardial release of MCP-1 is also suppressed in patients post-MI and in the presence of coronary artery disease. Thirdly, and perhaps most importantly, the human release pattern of cytokines sometimes reflects the pattern seen in rodent models, but in other cases it does not. This has significance for the progression of all translational research from murine studies.

Several authors have described different findings from ours; in particular, they have described increased SDF1a expression following MI [2,4,5]. However, we believe that this may be due to differences in strain of mouse used (CD1 mice in [2]) or species studied (rats in [4,5]). In contrast, we used C57Bl6 mice in our studies and found a decrease in RNA and protein expression. However, discrepancies between strains and species lend weight to our conclusions that interspecies variations are significant, and that observational translational clinical trials are paramount before progressing to clinical trials of therapies based on rodent data.

SDF-1a has been shown to be chemoattractant for stem cells [2,4,23], pro-angiogenic [24] and anti-apoptotic for cardiac myocytes [25]. Whilst the stem cell chemotactic properties capture the imagination, the absence of release of SDF-1a from the human heart suggest that this may not be its primary mechanism of action following MI. Confounding the issue, and further emphasizing the difference between rodent models and man, is the presence of coronary artery disease in humans. The suppression of SDF-1a release from the heart in both stable and unstable coronary artery syndromes suggests that coronary artery disease itself reduces the release of SDF-1a. Perhaps the coronary artery plaques themselves sequester SDF-1a from the circulation. The mechanism of SDF-1a inducing cardiac repair remains incompletely understood, but our data demonstrates that in humans, SDF-1a release from the heart is reduced after MI. Some prior observational studies have shown increased peripheral blood levels of SDF-1a after MI [8,9] but our data would suggest that if elevated levels of SDF-1a are detected in peripheral blood, it originates from tissues other than the heart.

It is not known whether the reduction in cytokine release of SDF-1a and MCP-1 are beneficial or detrimental to the LV remodeling process. In the case of SDF-1a, we and others [1,7] have shown that there is reduced myocardial SDF-1a production, and there is experimental evidence that myocardial delivery of SDF-1a can repair damaged myocardium [25]. With our human data mimicking the murine data, it would be reasonable to speculate that SDF-1a delivery to damaged human myocardium would result in improved LV function. However, with respect to MCP-1, quite the opposite is true. Elevated levels of MCP-1 have been demonstrated in peripheral blood following MI [18-20] and higher levels correlate with poorer prognosis [21]. The animal model shows increased MCP-1 RNA in the hearts of rodents subjected to MI, and one might logically speculate that the elevated peripheral blood level of MCP-1 in humans after MI is produced by, and released from, the damaged heart tissue. A knockout animal model has shown that deletion of MCP-1 has a beneficial effect on post-infarction LV remodeling [26]. Extrapolating from all this, one might expect a strategy of MCP-1 antagonism after MI to be therapeutically effective. However, our data shows that there is no increased release of MCP-1 from the human heart following MI, in fact there is a decrease. Therefore, it would likely not be appropriate to pursue blockade of myocardial MCP-1 based on our current results. This underscores the importance of human observational clinical studies to ensure that the animal model is actually reflective of the human disease. Performing this step in the advancement from the bench to the bedside will prevent costly studies that, although reasonable based on animal study data, are unlikely to succeed based on the human data. This will avoid exposing patients to unnecessary risk and may save considerable amounts for ever-shrinking research budgets.

For all who study animal models of human disease, it is imperative to acknowledge that all models have limitations. While it is easy and relatively inexpensive to perform quantitative studies on murine cardiac mRNA, it is comparatively difficult and expensive to perform such studies on human cardiac tissue. Furthermore, there are ethical and logistic difficulties in procuring human heart samples. Thus, we frequently presume that the murine findings will apply to the human situation; our results suggest that this may not be the case. A limitation to our study is that we have not directly compared human mRNA to murine mRNA. This is because of the aforementioned difficulties in procuring human heart tissue. We also did not directly compare the transmyocardial release of these cytokines between species, because the coronary sinus in the mouse is simply too small to access. We therefore chose the most appropriate available samples to compare between species, but our results should be interpreted with these limitations in mind. Another limitation of our study is that there were more males in the 3 coronary artery disease groups than in the normal control group (which is reflective of coronary artery disease's predilection for male subjects); therefore we cannot exclude gender differences as playing a small role in the differences between groups. However, we used all male mice in the murine study and showed similar response to MI with SDF-1a, but different response with MCP-1. This suggests that gender differences are less likely to have confounded our clinical results, but we cannot fully exclude the possibility of a gender effect in cytokine release profiles. This possibility warrants further study.

## Conclusions

Release of SDF-1a from the heart into the circulation following MI is suppressed in humans, and this is similar to the murine model. Release of MCP-1 is also suppressed, but this is in direct contra-distinction to the murine model. Observational clinical studies like this are important to confirm whether the human disease state is accurately reflected by the animal model, and to guide future therapeutic strategies.

## List of Abbreviations Used

SDF-1a: Stromal-derived factor 1-alpha; MCP-1: Monocyte chemotactic protein 1; CCR2: C-C chemokine receptor 2; CXCR4: CXC chemokine receptor 4; MI: myocardial infarction; LV: left ventricle; KCl: potassium chloride; mRNA: messenger ribonucleic acid.

## Competing interests

The authors declare that they have no competing interests.

## Authors' contributions

AB conceived the study, collected human samples, performed animal experiments, data interpretation and manuscript preparation. YY was involved in study design, data analysis and manuscript preparation. HS performed animal studies, RNA studies and manuscript preparation. JH performed animal studies, RNA studies and data analysis. JY performed animal studies, RNA studies and data analysis. JP collected patient data and samples, data analysis. RS animal studies and data analysis. DZ performed animal studies and data analysis. DP collected patient data and samples, data analysis. CK performed ELISAs and data analysis. RW collected human samples, data analysis and manuscript preparation. AJ performed ELISAs, study design and data analysis. AW conceived of study, collected human samples, data analysis and manuscript preparation. All authors read and approved the final manuscript.

## References

[B1] Wei Y-j Tang Y Li J Cui C-j Zhang H Zhang X-l Zhang H Hu S-s Cloning and expression pattern of dog SDF-1 and the implications of altered expression of SDF-1 in ischemic myocardium Cytokine 2007 40 52 59 10.1016/j.cyto.2007.08.004 17910919

[B2] Abbott JD Huang Y Liu D Hickey R Krause DS Giordano FJ Stromal Cell-Derived Factor-1{alpha} Plays a Critical Role in Stem Cell Recruitment to the Heart After Myocardial Infarction but Is Not Sufficient to Induce Homing in the Absence of Injury Circulation 2004 110 3300 3305 10.1161/01.CIR.0000147780.30124.CF 15533866

[B3] Vandervelde S van Luyn MJA Tio RA Harmsen MC Signaling factors in stem cell-mediated repair of infarcted myocardium Journal of Molecular and Cellular Cardiology 2005 39 363 376 10.1016/j.yjmcc.2005.05.012 15992820

[B4] Askari AT Unzek S Popovic ZB Goldman CK Forudi F Kiedrowski M Rovner A Ellis SG Thomas JD DiCorleto PE Effect of stromal-cell-derived factor 1 on stem-cell homing and tissue regeneration in ischaemic cardiomyopathy The Lancet 2003 362 697 703 10.1016/S0140-6736(03)14232-8 12957092

[B5] Pillarisetti K Gupta S Cloning and Relative Expression Analysis of Rat Stromal Cell Derived Factor-1 (SDF-1): SDF-1 α mRNA Is Selectively Induced in Rat Model of Myocardial Infarction Inflammation 2001 25 293 300 10.1023/A:1012808525370 11820456

[B6] Ma J Ge J Zhang S Sun A Shen J Chen L Wang K Zou Y Time course of myocardial stromal cell-derived factor 1 expression and beneficial effects of intravenously administered bone marrow stem cells in rats with experimental myocardial infarction Basic Research in Cardiology 2005 100 217 223 10.1007/s00395-005-0521-z 15754085

[B7] Vandervelde S van Luyn MJA Rozenbaum MH Petersen AH Tio RA Harmsen MC Stem cell-related cardiac gene expression early after murine myocardial infarction Cardiovascular Research 2007 73 783 793 10.1016/j.cardiores.2006.11.030 17208206

[B8] Lee BC Hsu HC Tseng WYI Su MYM Chen SY Wu YW Chien KL Chen MF Effect of cardiac rehabilitation on angiogenic cytokines in postinfarction patients Heart 2009 95 1012 1018 10.1136/hrt.2008.153510 19304668

[B9] Wang Y Johnsen HE Mortensen S Bindslev L Sejersten Ripa R Haack-Sorensen M Jorgensen E Fang W Kastrup J Changes in circulating mesenchymal stem cells, stem cell homing factor, and vascular growth factors in patients with acute ST elevation myocardial infarction treated with primary percutaneous coronary intervention Heart 2006 92 768 774 16251230 10.1136/hrt.2005.069799 PMC1860647

[B10] Leone AM Rutella S Bonanno G Contemi AM de Ritis DG Giannico MB Rebuzzi AG Leone G Crea F Endogenous G-CSF and CD34+ cell mobilization after acute myocardial infarction International Journal of Cardiology 2006 111 202 208 10.1016/j.ijcard.2005.06.043 16051386

[B11] Gu L Okada Y Clinton SK Gerard C Sukhova GK Libby P Rollins BJ Absence of Monocyte Chemoattractant Protein-1 Reduces Atherosclerosis in Low Density Lipoprotein Receptor-Deficient Mice Molecular Cell 1998 2 275 281 10.1016/S1097-2765(00)80139-2 9734366

[B12] Zhong L Chen WQ Ji XP Zhang M Zhao YX Yao GH Zhang PF Zhang C Zhang Y Dominant-negative mutation of monocyte chemoattractant protein-1 prevents vulnerable plaques from rupture in rabbits independent of serum lipid levels Journal of Cellular and Molecular Medicine 2008 12 2362 2371 10.1111/j.1582-4934.2008.00261.x 18266972 PMC4514114

[B13] Birdsall HH Green DM Trial J Youker KA Burns AR MacKay CR LaRosa GJ Hawkins HK Smith CW Michael LH Complement C5a, TGF-fl1, and MCP-1, in Sequence, Induce Migration of Monocytes Into Ischemic Canine Myocardium Within the First One to Five Hours After Reperfusion Circulation 1997 95 684 692 9024158 10.1161/01.cir.95.3.684

[B14] Hayashidani S Tsutsui H Shiomi T Ikeuchi M Matsusaka H Suematsu N Wen J Egashira K Takeshita A Anti-Monocyte Chemoattractant Protein-1 Gene Therapy Attenuates Left Ventricular Remodeling and Failure After Experimental Myocardial Infarction Circulation 2003 108 2134 2140 10.1161/01.CIR.0000092890.29552.22 14517168

[B15] Zhang F Tsai S Kato K Yamanouchi D Wang C Rafii S Liu B Kent KC Transforming Growth Factor-beta Promotes Recruitment of Bone Marrow Cells and Bone Marrow-derived Mesenchymal Stem Cells through Stimulation of MCP-1 Production in Vascular Smooth Muscle Cells Journal of Biological Chemistry 2009 284 17564 17574 10.1074/jbc.M109.013987 19406748 PMC2719395

[B16] Xu F Shi J Yu B Ni W Wu X Gu Z Chemokines mediate mesenchymal stem cell migration toward gliomas in vitro Oncology Reports 2010 23 1561 1567 20428810 10.3892/or_00000796

[B17] Stumpf C Seybold K Petzi S Wasmeier G Raaz D Yilmaz A Anger T Daniel WG Garlichs CD Interleukin-10 improves left ventricular function in rats with heart failure subsequent to myocardial infarction European Journal of Heart Failure 2008 10 733 739 10.1016/j.ejheart.2008.06.007 18599346

[B18] Arakelyan A Petrkova J Hermanova Z Boyajyan A Lukl J Petrek M Serum Levels of the MCP-1 Chemokine in Patients With Ischemic Stroke and Myocardial Infarction Mediators of Inflammation 2005 2005 175 179 10.1155/MI.2005.175 16106105 PMC1526470

[B19] Murakami Y Kurosaki K Matsui K Shimada K Ikeda U Serum MCP-1 and VEGF Levels are not Affected by Inhibition of the Renin-Angiotensin System in Patients with Acute Myocardial Infarction Cardiovascular Drugs and Therapy 2003 17 249 255 10.1023/A:1026128308440 14574083

[B20] Matsumori A Furukawa Y Hashimoto T Yoshida A Ono K Shioi T Okada M Iwasaki A Nishio R Matsushima K Sasayama S Plasma levels of the monocyte chemotactic and activating factor/monocyte chemoattractant protein-1 are elevated in patients with acute myocardial infarction J Mol Cell Cardiol 1997 29 419 423 10.1006/jmcc.1996.0285 9040055

[B21] de Lemos JA Morrow DA Sabatine MS Murphy SA Gibson CM Antman EM McCabe CH Cannon CP Braunwald E Association Between Plasma Levels of Monocyte Chemoattractant Protein-1 and Long-Term Clinical Outcomes in Patients With Acute Coronary Syndromes Circulation 2003 107 690 695 10.1161/01.CIR.0000049742.68848.99 12578870

[B22] Yeghiazarians Y Zhang Y Prasad M Shih H Saini SA Takagawa J Sievers RE Wong ML Kapasi NK Mirsky R Injection of Bone Marrow Cell Extract Into Infarcted Hearts Results in Functional Improvement Comparable to Intact Cell Therapy Molecular Therapy 2009 17 1250 1256 10.1038/mt.2009.85 19384293 PMC2835212

[B23] Aiuti A Webb IJ Bleul CC Springer T Guttierez-Ramos JC The chemokine SDF-1 is a chemoattractant for human CD34+ hematopoietic progenitor cells and provides a new mechanism to explain the mobilization of CD34+ progenitors to peripheral blood Journal of Experimental Medicine 1997 185 111 120 10.1084/jem.185.1.111 8996247 PMC2196104

[B24] Elmadbouh I Haider HK Jiang S Idris NM Lu G Ashraf M Ex vivo delivered stromal cell-derived factor-1[alpha] promotes stem cell homing and induces angiomyogenesis in the infarcted myocardium Journal of Molecular and Cellular Cardiology 2007 42 792 803 10.1016/j.yjmcc.2007.02.001 17350033 PMC2753220

[B25] Saxena A Fish JE White MD Yu S Smyth JWP Shaw RM DiMaio JM Srivastava D Stromal Cell-Derived Factor-1{alpha} Is Cardioprotective After Myocardial Infarction Circulation 2008 117 2224 2231 10.1161/CIRCULATIONAHA.107.694992 18427137 PMC2743260

[B26] Frangogiannis NG Dewald O Xia Y Ren G Haudek S Leucker T Kraemer D Taffet G Rollins BJ Entman ML Critical Role of Monocyte Chemoattractant Protein-1/CC Chemokine Ligand 2 in the Pathogenesis of Ischemic Cardiomyopathy Circulation 2007 115 584 592 10.1161/CIRCULATIONAHA.106.646091 17283277

